# Feasibility of a Pre-Operative Morphofunctional Assessment and the Effect of an Intervention Program with Oral Nutritional Supplements and Physical Exercise

**DOI:** 10.3390/nu17091509

**Published:** 2025-04-29

**Authors:** Natalia Mudarra-García, Fernando Roque-Rojas, Almudena Nieto-Ramos, Visitación Izquierdo-Izquierdo, Francisco Javier García-Sánchez

**Affiliations:** 1 Research Nursing Area, Hospital Universitario Ramón y Cajal, Instituto Ramón y Cajal de Investigación Sanitaria (IRYCIS), 28034 Madrid, Spain; nmudarra@enf.ucm.es; 2 Nursing Department, Faculty of Nurse, Phisiotherapy and Podology, University Complutense of Madrid, 28040 Madrid, Spain; 3 Surgical Prehabilitation Unit, Hospital Universitario Infanta Cristina, Instituto de Investigación Sanitaria Hospital Puerta de Hierro Segovia Arana (IDIPHISA), 28981 Madrid, Spain; fernando.roque@salud.madrid.org (F.R.-R.); almudena.nieto@salud.madrid.org (A.N.-R.); visitacion.izquierdo@salud.madrid.org (V.I.-I.); 4 Medical Department, Faculty of Medicine, University Complutense of Madrid, 28040 Madrid, Spain

**Keywords:** surgical prehabilitation, morphofuntional assessment, oral nutrient supplementation, disease-related malnutrition

## Abstract

**Background:** Surgical patients often experience nutritional imbalances due to their underlying condition and the forthcoming surgical procedure. These imbalances can increase the risk of post-operative complications. To mitigate such risks, a comprehensive nutritional assessment—also known as morphofunctional assessment—should be conducted. This includes evaluating body composition (muscle and fat mass), muscle strength, and functional capacity. **Methods:** We conducted an observational, prospective, pre-post interventional study involving 138 patients undergoing major elective surgery. Each patient received a morphofunctional assessment and an individualized prehabilitation program, including nutritional supplementation, physical exercise, and comorbidity optimization for 21 days before surgery and one month afterward. Outcomes were assessed through bioimpedance (muscle mass), muscle ultrasound (QRF thickness), dynamometry (strength), and visceral fat ultrasound (fat reduction). **Results:** The patient’s morphofunctional assessment and subsequent nutritional and physical exercise optimization performed during the month before surgery in the prehabilitation consultation led to an increase in muscle mass (measured by bioimpedance analysis, *p* = 0.001), and muscle ultrasound, (QRF thickness: *p* < 0.001) and dinamometry (muscle strength: *p* = 0.014); a reduction in preperitoneal visceral fat thickness (reduction *p* < 0.001); and an improvement in the patients’ nutritional status, with a decrease in malnutrition rates (64.8% vs. 31.8%). As a result, post-operative complications were effectively prevented (*p* < 0.001). **Conclusions:** Pre-operative patient optimization by means of a prehabilitation program led to increased muscle strength, improved muscle mass, reduced complication rates, and shorter hospital stays. In addition, patients maintained their quality of life and functional capacity following surgery.

## 1. Introduction

Surgical patients frequently present with nutritional imbalances caused by the underlying disease that necessitates hospitalization and the subsequent surgical intervention. These imbalances, combined with perioperative fasting and inflammatory responses, may lead to complications such as delayed recovery, infections, or poor wound healing. Several factors contribute to the onset of malnutrition in surgical patients, including advanced age, chronic comorbidities, oncological conditions, and poor oral intake [1]. This comprehensive approach, termed morphofunctional assessment, includes evaluation of body composition (via bioimpedance and ultrasound), muscle strength (dynamometry), and physical functionality (Barthel Index), offering a multidimensional picture of the patient’s nutritional and functional status [2,3].

Malnutrition in this context is associated with increased morbidity and mortality, longer hospital stays, higher readmission rates, and increased healthcare costs. A Spanish multicenter study reported complication rates exceeding 40% in malnourished surgical patients compared to 18% in well-nourished ones [4].

Surgical patients tend to experience abnormalities in nutritional status due to the underlying pathology that requires hospitalization and the subsequent surgical process. This may lead to post-operative complications, potentially creating a vicious cycle [1]. The most important factors that significantly contribute to the development of malnutrition in surgical patients include age, pre-existing chronic comorbidities, oncologic conditions, and prolonged peri-operative fasting periods [4].

There are several critical points during the peri-operative period when a patient’s nutritional status and body composition may be compromised. Both the underlying disease and pre-operative treatment can lead to metabolic disturbances and inflammatory processes that alter body composition. In addition, patients may struggle to meet their nutritional requirements through a regular diet, ultimately facing surgery in a state of nutritional deficiency, which significantly impacts the post-operative period [5]. A malnourished patient shows changes in body composition, with a marked decline in functional capacity and deterioration in immune and cardiorespiratory function. Various studies have revealed that the consequences of such malnutrition negatively impact patient recovery, leading to higher morbidity and mortality rates, increased hospital stays with a higher rate of readmission, and consequently, greater healthcare costs [6,7].

To optimize patients during the pre-operative period and enhance their functional capacity while reducing post-operative complications, it is recommended that they undergo a prehabilitation period. This should include physical improvement, cognitive intervention, smoking cessation, anemia correction, and nutritional assessment and intervention [7,8].

From a nutritional perspective, all patients scheduled for major surgery should undergo nutritional screening and a comprehensive assessment, followed by the establishment of a nutritional treatment plan with monitoring of tolerance and adherence. In addition, it is recommended that all patients at nutritional risk or already malnourished receive pre-operative nutritional treatment for at least seven to ten days before surgery [9]. However, to ensure an appropriate nutritional approach, nutrition should be fully assessed, including a detailed analysis of body composition, particularly muscle mass.

Body composition analysis using bioelectrical impedance analysis (BIA) has proven useful in assessing short- and long-term changes in body composition, nutritional prognosis, and as an indicator of morbidity and mortality risk. Another clinically feasible technique for assessing muscle mass is ultrasound, which provides a detailed analysis of muscle morphology. Specifically, assessing the rectus femoris muscle via ultrasonography could serve as a valuable tool for determining muscle mass in pre-operative patients [3,10,11].

Muscle strength assessment is also crucial. According to the European Working Group on Sarcopenia in Older People (EWGSOP2), low muscle strength combined with reduced muscle mass confirms a diagnosis of sarcopenia, with the severity determined by impaired muscle performance [12].

Therefore, assessing muscle mass using ultrasound and muscle strength through dynamometry is essential to confirm the diagnosis of sarcopenia in surgical patients. Moreover, these assessments should be integrated into routine clinical practice as part of the nutritional evaluation in surgical candidates. Furthermore, it may be possible to extrapolate this condition observed in older adults to the pre-operative patient population, enabling the implementation of targeted interventions aimed at optimizing their status in preparation for surgery.

Despite growing evidence, morphofunctional assessment is not routinely implemented in clinical surgical pathways. This study aimed to evaluate the feasibility and clinical impact of a pre-operative optimization protocol based on morphofunctional assessment and individualized intervention. The intervention included oral nutritional supplementation, personalized exercise plans, and comorbidity correction.

## 2. Materials and Methods

### 2.1. Study Design

We conducted a prospective pre–post interventional study between January and December 2022 at Infanta Cristina University Hospital in Parla (Madrid, Spain). The study was approved by the Ethics Committee of the Instituto de Investigación Biomédica Segovia-Arana of Puerta de Hierro University Hospital (Approval Code: CI 22/22, 20 February 2022), and was carried out in accordance with the Declaration of Helsinki.

Additionally, a historical control group was used for comparative purposes. This group comprised 76 consecutive patients who underwent similar major elective surgeries at the same hospital during the year 2019, before the implementation of the prehabilitation consultation. These patients did not receive any pre-operative optimization. Data for this group were obtained from a prior observational study conducted at the same institution and matched by age, type of surgery, and comorbidities to the intervention group. This comparison allows for contextualizing the potential impact of the prehabilitation program.

Written informed consent was obtained from each participant. To ensure anonymity and confidentiality, patient data were coded and stored in secure, password-protected databases accessible only to authorized research personnel, in compliance with Spanish legislation (Organic Law 3/2018 and RD 1090/2015).

### 2.2. Study Population

Patients referred to the Surgical Prehabilitation Unit were considered eligible if they met the following criteria.

#### 2.2.1. Inclusion Criteria

- Participants aged 18 years or older.
- Referred for major elective surgery requiring hospital admission (e.g., oncologic resections such as mastectomy, colon resection, nephrectomy, hysterectomy), typically involving general anesthesia and moderate to high morbidity risk, capable of understanding and consenting to the study.
- Physically and mentally able to complete assessments.

#### 2.2.2. Exclusion Criteria

- Inability to eat orally.
- Transfer to another hospital prior to surgery.
- Refusal to sign informed consent.

Referral indications were based on surgical complexity and patient risk profile; while not limited to high-risk patients, the majority had oncologic diagnoses.

We acknowledge that excluding patients unable to eat orally could introduce selection bias, and this is considered in the discussion of limitations.

### 2.3. Sample Size

Based on the total number of patients seen in the prehabilitation unit with the previously described characteristics (*n* = 150), and assuming that all of them would undergo the indicated assessment, a sample size of 138 patients was calculated. This estimation was based on a 95% confidence level, a 3% margin of error, and an expected proportion of 5%.

### 2.4. Studied Variables

Clinical variables: age (years); sex (male/female); type of pathology (breast cancer IDC, colorectal, renal adenocarcinoma, endometrial adenocarcinoma); type of procedure (tumorectomy, mastectomy, colon resection, nephrectomy, hysterectomy); active malignancy oncologic patient (yes/no); neoadjuvant treatment (yes/no).

Anthropometric measurements:

- Height: measured with a calibrated wall-mounted stadiometer (m).
- Weight: measured using a calibrated clinical digital scale (kg).
- BMI calculated as weight (kg)/height^2^ (m^2^).

Patients were assessed in light clothing, without shoes.

Biochemical variables:

- Albumin (g/dL); prealbumin (mg/dL); proteins (g/dL); total cholesterol (mg/dL); lymphocytes (units/mm^3^).
- Inflammation and anemia indicators were monitored in clinical practice (e.g., CRP, hemoglobin), though not all were included in the final analysis.

Muscle strength:

- Assessed using a JAMAR Hydraulic Hand Dynamometer (Model J00105).
- Test conducted with the dominant hand, seated with elbow at 90°, patients were instructed to exert maximum force, and three attempts were recorded with a 30-s rest interval; the average value was calculated (kg).

Body composition:

- Bioelectrical Impedance Analysis (BIA) using Beurer BF 1000 (consumer-grade device).
- Muscle mass (%) and fat mass (%) estimated.
- Patients were measured fasting, without recent physical activity, and after urination.

While we acknowledge this is not a medical-grade device, efforts were made to take measurements.

**Figure 1:**
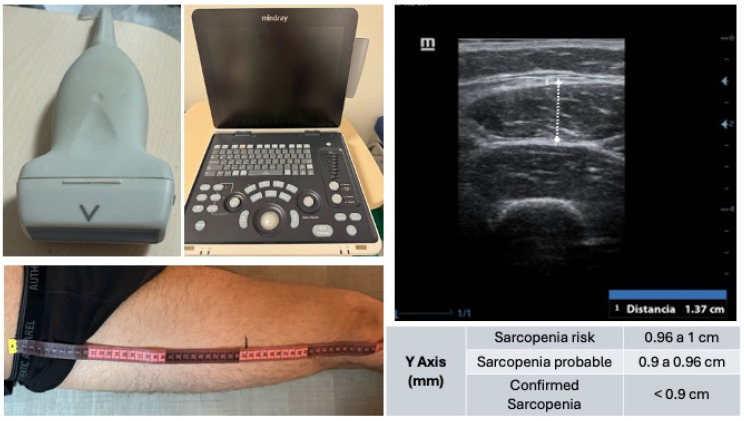
Ultrasound assessment of the rectus femoris muscle for sarcopenia screening. Shown (top left) is the linear probe (10–12 MHz) used for image acquisition, alongside the portable ultrasound system Mindray Z60 (center). The ultrasound image (top right) corresponds to a transverse view at the distal third of the thigh, identifying from superficial to deep: ultrasound gel, skin, subcutaneous tissue, vastus lateralis muscle, rectus femoris muscle, and femur. The bottom left image depicts the anatomical landmarking process for measurement, based on the distance between the anterior superior iliac spine and the upper edge of the patella. Reference values for the Y-axis measurement of the rectus femoris are included, used to classify sarcopenia risk and diagnosis.

Muscle ultrasound:

- Device: Mindray Z60 with a 10–12 MHz linear probe.
- Rectus femoris (QRF) was measured in the dominant leg, supine position, with the probe perpendicular and placed at the lower third of the thigh (measured from the anterior superior iliac spine to the upper border of the patella). Analysis was performed without compression, identifying the QRF by its central tendon, and measuring the anteroposterior thickness of the QRF (Y-axis, cm). The Y-axis index (Y-axis/m^2^) was also calculated (Figure 1).
- Image depth: 4 cm, measured without compression.

One experienced operator performed all assessments; interobserver variability was not assessed.

Visceral fat ultrasound:

- Device: Mindray Z60 with a 10–12 MHz linear probe.
- Measured preperitoneal fat at the midline between xiphoid and umbilicus. The preperitoneal visceral fat compartment was identified between the inner surface of the linea alba and the peritoneal membrane. Measured during unforced expiration. Three measurements were taken and averaged.
- No bladder filling was required.

Nutritional status:

- The GLIM criteria were applied, assessing phenotypic criteria (unintentional weight loss > 5% in 6 months, BMI < 18.5 kg/m^2^) and etiologic criteria such as inflammation (elevated C-reactive protein), inflammatory disease, or a reduction in food intake greater than 50%.

**Functionality and quality of life:**

- Barthel Index, original 10-item version, assessed by trained nurses.
- EuroQoL-5D-5L version (no VAS), validated in Spanish.

Complications:

- Post-operative complications were recorded, including general complications (yes/no), infectious complications (yes/no), suture dehiscence (yes/no), surgical wound complications (yes/no), and surgical wound complications at two weeks (yes/no).
- Complications were assessed at 14 and 30 days post-operatively.
- No readmissions beyond 30 days were included.

### 2.5. Intervention

#### 2.5.1. Overview of Prehabilitation Program

All patients attended a prehabilitation consultation conducted by an Advanced Practice Nurse (APN) within 48 h after inclusion on the surgical waiting list. The program was designed to optimize nutritional status, physical function, and comorbidity control over the 21 days prior to surgery and extended until one month post-operatively.

The APN led the intervention and performed all evaluations and follow-ups, in collaboration with an internist for medical and nutritional prescription. Other specialists (e.g., psychologists, physiotherapists) were consulted only when needed.

#### 2.5.2. Initial Assessment

During the first consultation, a comprehensive morphofunctional assessment was conducted, including:

- Nutritional screening using GLIM criteria.
- Body composition analysis (bioimpedance and ultrasound).
- Muscle strength assessment (dynamometry).
- Functional status (Barthel Index).
- Quality of life (EQ-5D-5L).
- Biochemical analysis (including albumin, prealbumin, cholesterol, CRP, hemoglobin, lymphocytes).
- Psychological evaluation (see below).

#### 2.5.3. Psychological Assessment

- Identified deficit related to self-esteem and body image employing Rosenberg Scale in active malignant oncology patients and BIS scale to all patients scheduled for surgery that may result in changes to body image, such as mastectomy or stoma formation.
- If any alterations are identified in these evaluations, a referral to a psycho-oncologist is made.

#### 2.5.4. Exercise Intervention

All patients received individualized counseling and a personalized physical exercise plan (based on the Vivifrail program). The physical exercise is tailored to the patient’s age, frailty criteria, and baseline functional status.

- At least 150 min per week of combined aerobic (based RPE) and resistance training (home-based).
- Daily use of incentive spirometer for respiratory training (model: Spiro-Ball).

#### 2.5.5. Nutritional Intervention

All patients received oral nutritional supplements (ONS) according to their nutritional status and comorbidities. Products were from PERSAN FARMA S.A. and included:

1. Overweight or Sarcopenic Obesity.
  - Diet: protein-rich (>71 g/day), normocaloric, mediterranean food-based.
  - Supplement: NUTAVANT HP^®^, 2 bottles/day (250 mL each). Per 100 mL: 108 kcal, 6 g protein, 12 g carbs, 4 g fat (Table A1).
2. Mixed malnutrition or Disease-Related Malnutrition (DRM).
  Characterized by the concurrent presence of both sarcopenia and excess adiposity, is particularly challenging in surgical patients due to its association with adverse metabolic and inflammatory profiles.
  - Diet: high-protein and high-calorie.
  - Supplement: NUTAVANT PLUS^®^, 2 bottles/day. Per 100 mL: 162 kcal, 8.9 g protein, 16.9 g carbs, 6.5 g fat (Table A2).
3. Malnutrition with Carbohydrate Metabolism Alterations
  - Diet: low glycemic index, increased monounsaturated fat, higher fiber.
  - Supplement: NUTAVANT PLUS DIABETIC^®^, 2 bottles/day. Per 100 mL: 120 kcal, 6.6 g protein, 12 g carbs, 4.6 g fat, 1.8 g soluble fiber (Table A3).

Medical nutritional therapy (oral nutritional supplementation [ONS], prescribed by the internist associated with the prehabilitation consultation, to be taken for 21 days before surgery and one month after).

Patients were instructed to consume ONS between meals or at bedtime to avoid appetite suppression during main meals. Micronutrient deficiencies (e.g., vitamin D, iron) were addressed when detected in labs, but not supplemented routinely.

The process was optimized for three weeks before surgery and one month postoperatively. Patients received tailored nutritional education and therapy. This included tailored diets and nutritional supplementation with macronutrients and micronutrients, according to expert recommendations. Notably, immunonutrient-enriched diets were not used, as per the available scientific evidence on pre-operative nutritional supplementation.

#### 2.5.6. Follow-Up Visits and Adherence Monitoring

The second visit (day before surgery): A reassessment of all variables including nutritional status (providing support for the evaluation of adherence to dietary recommendations and prescribed physical exercise), performing the same evaluations as in the initial visit.

The third visit (one month after surgery): A complete reevaluation of nutritional status, quality of life, and functionality.

The adherence (defined as >80% adherence) was assessed through structured interviews and self-reported logs with both the patient and their accompanying caregiver, and the reported adherence levels were subsequently compared with objective outcomes obtained from the morphofunctional evaluation.

### 2.6. Statistical Analysis

Statistical analyses were performed using SPSS v27 software package (IBM, Armonk, NY, USA). A descriptive analysis was conducted for all study variables to examine their distribution. Categorical variables were reported using percentages for each response option. Quantitative variables were presented as mean and standard deviation. For comparisons between variables and hypothesis testing, the following statistical tests were used: Chi-square test for categorical variables; Student’s *t*-test for quantitative variables.

## 3. Results

### 3.1. Sample Characteristics

**Figure 2:**
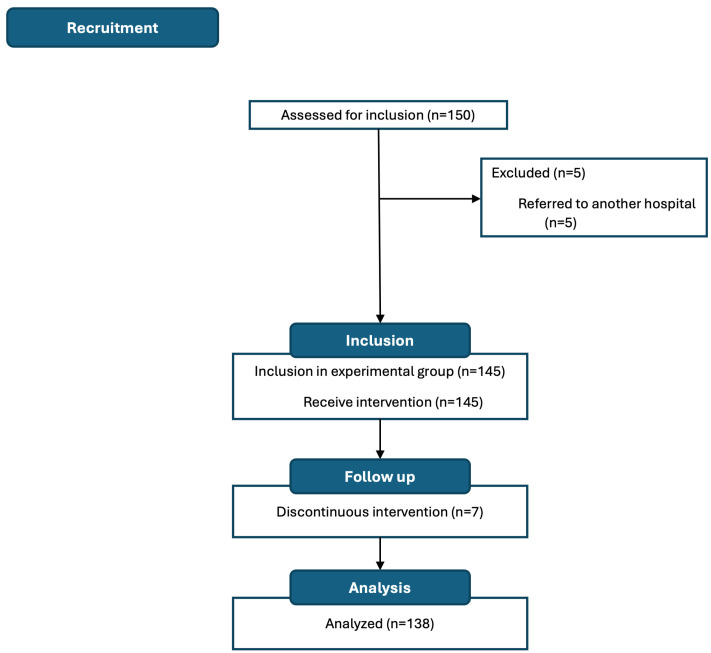
Study flow chart.

A total of 150 patients were recruited, of whom 138 (92.0%) were analyzed (Figure 2). Most patients were women (*n* = 84, 60.9%) with a mean age (SD) of 61.69 (13.22) years. The most common surgical procedure was colon resection (*n* = 49, 35.7%), followed by mastectomy and hysterectomy (*n* = 28, 20.5% each), nephrectomy (*n* = 18, 13.2%), and lumpectomy (n = 15, 10.1%). Most patients were oncology cases (*n* = 104, 75.3%), and only 27.0% (*n* = 37) received neoadjuvant therapy (Table A4).

### 3.2. Nutritional Status

According to GLIM criteria, 81 patients (64.8%) had malnutrition before prehabilitation, with severe malnutrition in 49 (39.2%) of them. After optimization, this number decreased to 42 patients (31.8%), and one month after surgery, only 35 patients (26.7%) met the criteria for malnutrition (Table A4).

#### 3.2.1. Before Prehabilitation

- 64.8% were malnourished per GLIM criteria (Table A5).
- Mean muscle mass: 33.54 ± 6.60% (Table A5).
- Mean fat mass: 33.45 ± 10.23% (Table A5).
- Visceral fat thickness: 0.68 ± 0.32 cm (Table A5).

#### 3.2.2. After Optimization

- The malnutrition rate decreased to 31.8% (*p* < 0.001).
- Muscle mass increased to 36.33 ± 6.45% (*p* = 0.001) (Table A5).
- Fat mass decreased to 31.03 ± 9.61% (*p* = 0.047).
- Visceral fat decreased to 0.55 ± 0.24 cm (*p* < 0.001).

#### 3.2.3. One Month Post-Surgery

- Malnutrition further decreased to 26.7%.
- Muscle mass increased to 36.69 ± 6.63% (*p* < 0.001) (Table A6).
- Fat mass reduced to 30.28 ± 10.07% (*p* = 0.011).
- Visceral fat: 0.50 ± 0.21 cm (*p* < 0.001).

### 3.3. Muscle Strength and Ultrasound Markers

#### 3.3.1. Handgrip Strength

- Baseline: 24.06 ± 9.30 kg.
- After optimization: 26.91 ± 9.76 kg (*p* = 0.014) (Table A5).
- One month post-op: 27.21 ± 9.59 kg (*p* = 0.007) (Table A6).

#### 3.3.2. QRF Muscle Thickness (Y-Axis)

- Baseline: 1.16 ± 0.32 cm.
- After optimization: 1.40 ± 0.33 cm (*p* < 0.001) (Table A5).
- One month post-op: 1.37 ± 0.31 cm (*p* < 0.001) (Table A6).

#### 3.3.3. Adherence

The supplementation regimen was prescribed based on the type of malnutrition present and the daily intake requirements calculated. Most patients were prescribed two bottles of oral nutritional supplements (ONS) per day. Adherence with the prescribed ONS was high (*n* = 121, 87.6%) (Table A4). Only 8.69% consumed one bottle per day, while 3.6% did not take any.

At the first prehabilitation consultation, 71.0% of patients engaged in physical exercise. This increased to 94.2% after optimization (*p* < 0.001) (Table A5).

#### 3.3.4. Complications

Of the total patients, only 14.2% (*n* = 19) underwent post-operative complications, of which 6.0% (*n* = 8) were infectious. In regard to surgical wound complications at two weeks, only 2.3% (*n* = 3) showed abnormalities (Table A4).

In regard to the post-operative period, only 19 patients (14.2%) underwent complications, most of which were infectious (eight patients). It can be observed that patients who had complications had a lower percentage of muscle mass, a higher percentage of fat mass, a smaller Y-axis thickness of the QRF, a greater thickness of preperitoneal visceral fat, and lower muscle strength (Table A9).

### 3.4. Quality of Life and Functionality

In regard to quality of life and functionality, patients maintained similar scores across the three visits (Table A4 and Table A8). No significant reduction in functionality was observed during the peri-operative period, nor were there statistically significant differences between visits (Table A5 and Table A6). The scores in the different dimensions of the EuroQoL 5D test improved at the second visit, after prehabilitation, with no abnormalities observed in the categories one month after surgery in any patient (Table A8).

### 3.5. Length of Hospital Stay (LOS)

Patients who underwent surgery without participating in the prehabilitation program (control group, *n* = 76) had an average hospital stay of 11.63 days (SD: 10.63), whereas those who followed the prehabilitation protocol (intervention group, *n* = 138) had a significantly shorter average stay of 8.34 days (SD: 6.70) (*p* = 0.004) (Table A10).

### 3.6. Other Variables

In regard to biochemical variables, patients maintained similar levels across the three visits (Table A4). However, statistically significant differences were observed in albumin levels between the first visit and the visit one month after surgery (4.01 ± 1.95 g/dL vs. 3.45 ± 0.07 g/dL; *p* = 0.002) (Table A6).

## 4. Discussion

### 4.1. Principal Findings

This study reports the health outcomes of patients who underwent major elective surgery after taking part in a pre-operative optimization program led by an advanced practice nurse in collaboration with an internist associated with the project. The outcomes compared were those obtained at the time the patient was included in the SWL, after optimization, and during the month of surgery. In addition, post-operative complications were compared between patients treated in 2019—before the implementation of the surgical prehabilitation consultation (data obtained from a prior study at the hospital)—and those who underwent optimization (Table A10).

Programs involving multiple professionals can be ineffective due to delays in patient treatment and incorrect referrals [13]. The model used here requires only two healthcare professionals. Other models involve multiple specialists (such as hematologists, endocrinologists, cardiologists, pulmonologists, and physiotherapists) and require an average of five visits per patient [9]. In the protocol implemented at the Infanta Cristina University Hospital in Parla (Madrid), as reported in this paper, patient assessment, management, and care coordination are overseen by the advanced practice nurse in collaboration with the internist associated with the program. This enables the optimization process to commence within 48 h of adding the patient to the protocol. Empowering a nurse to take on roles traditionally performed by other physicians streamlines access to therapies and simplifies the treatment of complex patients [14].

In addition to pre-post comparisons within the intervention group, outcomes were contextualized by comparing them with a historical control group of patients operated on prior to the implementation of the prehabilitation program. While this provides a useful benchmark to estimate the potential impact of the intervention, the use of non-concurrent controls introduces potential bias and limits the strength of causal inferences. Nevertheless, the large difference in complication rates (52.6% vs. 14.2%) and LOS (11.63 vs. 8.34 days) suggests a clinically meaningful effect.

### 4.2. Interpretation in Context

Disease-related malnutrition (DRM) is associated with an imbalance between the patient’s intake and their energy and protein requirements, leading to metabolic and functional changes at the body level. There are multiple limitations to traditional nutritional assessment parameters, such as body mass index, weight loss, or traditional analytical parameters like albumin or lymphocytes. Therefore, we propose a new approach to nutritional assessment and management, focusing on the patient’s morphofunctional evaluation, assessing changes in body composition and function with new parameters using techniques such as bioimpedance, ultrasound, dynamometry, and functional tests [3].

Although our intervention was not focused on a specific dietary pattern, recent studies highlight the potential benefits of integrating the Mediterranean diet into perioperative nutritional strategies. Originally studied in the context of bariatric surgery, this dietary model has shown favorable effects on weight loss, visceral fat reduction, and cardiometabolic parameters, which are key targets in prehabilitation. Ruiz-Tovar et al. demonstrated that patients with better adherence to the Mediterranean diet achieved greater post-operative weight loss and improvements in lipid profiles [15]. Similarly, Gastaldo et al. reviewed evidence supporting the use of a Mediterranean dietary pattern before and after surgery to enhance recovery and reduce complications [16]. Given its anti-inflammatory and antioxidant properties, the Mediterranean diet may represent a valuable component of perioperative care across diverse surgical populations, not limited to metabolic surgery.

### 4.3. Clinical Implications

A systematic review of patients aged over 65 states that combining nutritional supplementation with physical exercise improves muscle strength, promotes mobility, and prevents sarcopenia. It also emphasizes that supplementation should be accompanied by individualized dietary guidelines [17]. This study draws the same conclusions as the cited systematic review, highlighting that our study population included all age groups, not just patients aged over 65. As reflected in the review, our study attained improvements in muscle strength measured by dynamometry (an increase of 2.85 kg), improvements in the Y-axis of the RF measured by ultrasound (an increase of 0.24 cm), an increase in muscle mass measured by bioimpedance (an improvement of 2.79%), a reduction in preperitoneal visceral fat tissue measured by abdominal ultrasound (a reduction of 0.13 cm), and nutritional assessment improvements based on GLIM criteria (64.8% of patients were malnourished before prehabilitation vs. 26.7% one month after intervention). This was attained by implementing optimization strategies (ONS, tailored diet, personalized exercise, correction of comorbidity disorders, and treatment adjustments) before surgery (Table A6).

Furthermore, this study included an assessment of patient functionality using the Barthel Index and quality of life using the EuroQol-5 dimensions scale. These measurements confirmed that the level of independence in activities of daily living and patients’ quality of life remained intact thanks to the optimization process (as reported in the sample, patients already showed good quality of life and independence, which remained unchanged one month after surgery) (Table A6).

Moreover, significant improvements were observed in the values recorded before the intervention and after optimization, compared to those obtained one month after surgery (a period during which patients continued following the given recommendations). These results indicate that improvements in body composition and nutritional status were not diminished post-surgery and even improved in some parameters (Table A6). Among the small number of patients who underwent complications, it was observed that they had lower muscle mass, higher preperitoneal visceral fat tissue, and lower muscle strength (Table A9). It is worth noting that the reduction in complications was clearly linked to the individualized optimization process, as revealed in the study. Our study population had an average BMI of 28.54, which led to the optimization of these patients with a tailored 1500-calorie diet along with standard optimization. The positive impact of these measures is reflected in the reduction of fat following the prescribed treatment (an average decrease of 2.42% in fat mass, as measured by bioimpedance). This reduction in fat facilitates surgical intervention and decreases complications (Table A5).

Furthermore, adherence to treatment, supplementation (high adherence of 87.6%, measured using a home nutritional adherence test), and physical exercise (71% of our patients did not exercise before optimization, while 94.2% did after) was attained by means of personalized telephone follow-ups, conducted from the first day of optimization. This type of intervention substantially improves treatment adherence, especially during perioperative periods, as reported by Pfeiffer et al. [18], the European Society for Patient Adherence [19], and the meta-analysis conducted by Conn and Ruppar [20]. Likewise, Van Exter et al. also found significant improvements in adherence and outcomes through the use of oral nutritional supplements (ONS) when implementing specific patient-focused interventions [21].

In addition, this study collated data from a previous study involving patients who underwent surgery without the benefit of the prehabilitation consultation, as it had not yet been implemented. Post-operative complications were compared between patients who underwent surgery in 2019 (who did not have access to prehabilitation consultation, *n* = 76) and patients in this study who did (*n* = 138). The complication rate for patients who did not receive prehabilitation was 52.6% (*n* = 40), compared to 14.2% (*n* = 19) for those who did (*p* < 0.001). Regarding wound dehiscence, complications occurred in 21.1% (*n* = 16) of non-optimized patients versus 3% (*n* = 4) (*p* = 0.005). The length of hospital stay for patients who did not have access to prehabilitation was 11.63 ± 10.63 days, compared to 8.34 ± 6.70 days for those who did attend consultation (*p* = 0.004) (Table A10). The results obtained in this study reveal that pre-operative optimization significantly reduces the frequency of immediate and long-term post-operative complications (*p* < 0.001). This is consistent with another clinical study on patients undergoing major abdominal surgery, in which the post-operative complication rate was 31% following pre-operative optimization and 62% in the control group without optimization [22]. In regard to suture dehiscence, it was less common among participants in the program than in the control group. This finding aligns with a meta-analysis published in 2021, which revealed that improving nutritional status reduces the incidence of suture dehiscence by 29% [23]. In addition, hyperproteic and hypercaloric oral supplementation with added vitamin D may also be associated with a reduction in post-operative complications [24]. This aligns with the conclusions of Perry et al. (2021), who performed a meta-analysis of 10 clinical studies involving 643 patients [23]. Their analysis found that post-operative complications decreased in the group receiving whey protein supplementation (22%) compared to the control group (32%) (*p* = 0.001) [25]. Therefore, it can be concluded that administering hyperproteic ONS improves muscle mass recovery and muscle trophism, which are essential for early mobilization of patients post-surgery.

### 4.4. Clinical Care Coordination and Cost-Efficiency

Several meta-analyses present varying conclusions regarding the reduction in hospital stay duration for patients taking part in pre-operative optimization programs. One such analysis of nine randomized clinical trials did not observe differences between patients who participated in a pre-operative optimization program and those who did not [26]. However, Lambert et al. (2021) reported in their study a reduction of an average of 1.78 days in hospital stays for patients who participated in pre-operative optimization programs compared to those who did not [27]. These results are consistent with those obtained in this study, where hospital admission in the intervention group was reduced by an average of 3.29 days compared to the control group with the consequent cost savings, as referenced in the manuscript by Mudarra et al. (2025) regarding the economic benefits of surgical prehabilitation [28].

### 4.5. Limitations

The outcomes of this research should be interpreted within the context of its limitations, which are typical of the study design. The level of motivation of a patient who voluntarily takes part in research may differ significantly from that of other patients. In regard to the comparison between patients who benefited from prehabilitation and those who did not, patients who received treatment in the consultation may have had different motivations for following the medical recommendations for pre-operative optimization compared to those in the control group.

The study has certain limitations:

- Single-center design, which may affect external validity.
- Lack of randomization and potential for selection bias (e.g., exclusion of patients unable to eat orally).
- Use of consumer-grade BIA device (though with standardization).
- No long-term follow-up beyond one month post-op.
- Psychological results were not quantified or analyzed statistically.
- No formal classification of complications (e.g., Clavien–Dindo).

The comparison with the control group is based on historical data from patients treated in 2019, prior to the implementation of the prehabilitation consultation. These patients were not randomly assigned, and although matched by surgical type and general risk profile, unmeasured confounding variables and differences in care pathways over time may have influenced the observed differences. This constitutes a methodological limitation that must be acknowledged when interpreting the results.

Despite its limitations, the study presents favorable results obtained through the implementation of a pre-operative optimization program led by a liaison nurse, aimed at reducing the number of post-operative complications and shortening hospitalization time following major elective surgery, as well as improving muscle mass. The results are consistent with those of other studies on similar interventions.

Although these designs have low internal validity, their external validity is high because they reflect routine clinical practice and the value of interventions that can be performed in this context.

### 4.6. Future Directions

This study opens several avenues for further investigation. First, future research should include randomized controlled trials to confirm the clinical efficacy of this prehabilitation model and reduce potential biases. Second, longer follow-up periods are needed to determine whether the observed improvements in muscle mass, functional status, and complication rates are sustained beyond the early post-operative period.

Additionally, cost-effectiveness analyses would be valuable to support the implementation of these programs in routine care and to evaluate their economic impact. The integration of digital tools for remote monitoring of adherence and outcomes could enhance scalability and accessibility.

Future studies should also explore the adaptation of this intervention to other surgical specialties, such as cardiovascular or orthopedic surgery, and assess whether similar benefits are observed. Furthermore, the potential role of Mediterranean diet-based nutritional strategies within prehabilitation protocols warrants investigation in a broader surgical population.

Lastly, the validation and implementation of simplified morphofunctional tools—such as portable ultrasound and digital grip strength assessment—could facilitate widespread use in diverse clinical settings, including those with limited resources.

Future studies should aim for multicenter randomized designs, longer follow-up periods, and inclusion of cost-effectiveness analyses.

## 5. Conclusions

This study demonstrates that a nurse-led prehabilitation program integrating morphofunctional assessment, oral nutritional supplementation, and tailored physical activity is both feasible and effective in surgical patients. The intervention significantly improved muscle mass, strength, and nutritional status while reducing post-operative complications and hospital stay.

These findings support the integration of structured, personalized prehabilitation into standard surgical pathways to enhance patient outcomes and reduce healthcare burden. 

## Data Availability

The original contributions presented in this study are included in the article. Further inquiries can be directed to the corresponding author.

## Abbreviations

The following abbreviations are used in this manuscript:

BIA	Bioelectrical impedance analysis
BMI	Body mass index
DRM	Disease-related malnutrition
IDC	Invasive ductal carcinoma
GLIM	Global Leadership Initiative on Malnutrition
LOS	Length of stay
ONS	Oral nutrition supplements
QRF	Quadriceps rectus femoris
SWL	Surgical waiting list

## Appendix A

**Table A1:** Nutritional information of NUTAVANT HP.

NUTAVANT HP	Per 100 mL
Energy (kJ)	454
Energy (kcal)	108
Total Fat (g)	4.0
Saturated Fat (g)	2.7
Carbohydrates (g)	12
Sugars (g)	5.2
Lactose (g)	0.27
Dietary Fiber (g)	0
Protein (g)	6.0
Salt (g)	0.28

**Table A2:** Nutritional information of NUTAVANT PLUS.

NUTAVANT PLUS	Per 100 mL
Energy (kJ)	679
Energy (kcal)	162
Total Fat (g)	6.50
Saturated Fat (g)	1.9
Carbohydrates (g)	16.9
Sugars (g)	1.7
Lactose (g)	0.52
Dietary Fiber (g)	0
Protein (g)	8.90
Salt (g)	0.24

**Table A3:** Nutritional information of NUTAVANT PLUS DIABETIC.

NUTAVANT PLUS DIABETIC	Per 100 mL
Energy (kJ)	503
Energy (kcal)	120
Total Fat (g)	4.70
Saturated Fat (g)	1.00
Carbohydrates (g)	12
Sugars (g)	2.50
Lactose (g)	0.28
Dietary Fiber (g)	1.8
Protein (g)	6.60
Salt (g)	0.28

**Table A4:** Sociodemographic and clinical variables obtained during the initial consultation, the day before surgery, and one month after surgery (standard deviation).

		Before Prehabilitation	Before Surgery	One Month from Surgery
Age (years)		61.69 (13.22)	61.69 (13.22)	61.69 (13.22)
Gender N (%)	Female	84 (60.90)	84 (60.90)	84 (60.90)
	Male	54 (39.10)	54 (39.10)	54 (39.10)
BMI (kg/m^2^) (SD)		28.54 (5.89)	28.07 (5.46)	30.29 (21.93)
	Underweight	7 (5.10)	6 (4.30)	4 (2.90)
BMI Categorized N (%)	Normal weight	43 (31.20)	38 (27.50)	46 (33.30)
	Overweight	88 (63.80)	94 (68.10)	88 (63.80)
Albumin (g/dL)		4.01 (1.95)	4.11 (2.25)	3.45 (0.07)
Prealbumin (mg/dL)		24.08 (6.89)	23.45 (5.21)	22.4 (5.15)
Proteins (mg/dL)		6.97 (0.51)	7.01 (0.50)	5.95 (1.34)
Cholesterol (mg/dL)		179.11 (43.84)	168.98 (37.87)	
Lymphocytes (uni/mm^3^)		1770.80 (762.55)	1672.42 (650.40)	1833.30 (450.9)
Malnutrition (GLIM) (%)		64.80	31.80	26.70
Dynamometry (kg)		24.06 (9.30)	26.91 (9.76)	27.21 (9.59)
Muscle mass (%)		33.54 (6.60)	36.33 (6.45)	36.69 (6.63)
Fat mass (%)		33.45 (10.23)	31.03 (9.61)	30.28 (10.07)
Y QRF (cm)		1.16 (0.32)	1.40 (0.33)	1.37 (0.31)
Y/height^2^		0.482 (0.156)	0.528 (0.174)	0.525 (0.187)
Preperitoneal fat thickness (cm)		0.68 (0.32)	0.55 (0.24)	0.50 (0.21)
Barthel index		96.9 (10.05)	97.31 (7.94)	96.98 (8.39)
ONS 100% Adherence			121 (87.60%)	
Total complications				14.20%
Infectious complications				6.00%
Suture dehiscence				3.00%
Surgical wound complications				2.90%
Wound at 2 weeks complications				2.30%

**Table A5:** Clinical variables comparing results before treatment and after optimization (standard deviation).

		Before Prehabilitation	Before Surgery	*p*
BMI (kg/m^2^)		28.54 (5.89)	28.07 (5.46)	0.501
	Underweight	7 (5.10)	6 (4.30)	0.675
	Normal weight	43 (31.2)	38 (27.50)	
BMI Categorized N (%)	Overweight	88 (63.80)	94 (68.10)	
Albumin (g/dL)		4.01 (1.95)	4.11 (2.25)	0.718
Prealbumin (mg/dL)		24.08 (6.89)	23.45 (5.21)	0.418
Proteins (g/dL)		6.97 (0.51)	7.01 (0.50)	0.478
Cholesterol (mg/dL)		179.11 (43.84)	168.98 (37.87)	0.054
Lymphocytes (uni/mm^3^)		1770.80 (762.55)	1672.42 (650.40)	0.265
Malnutrition (GLIM) (%)		64.80	31.80	<0.001
Dynamometry (kg)		24.06 (9.30)	26.91 (9.76)	0.014
Muscle mass (%)		33.54 (6.60)	36.33 (6.45)	0.001
Fat mass (%)		33.45 (10.23)	31.03 (9.61)	0.047
Y QRF (cm)		1.16 (0.32)	1.40 (0.33)	<0.001
Y/(height^2^)		0.482 (0.156)	0.528 (0.174)	0.023
Preperitoneal fat thickness (cm)		0.680 (0.32)	0.550 (0.24)	<0.001
Barthel index (SD)		96.90 (10.05)	97.31 (7.94)	0.719
Exercise Adherence (%)		71.00	94.00	<0.001

**Table A6:** Clinical variables comparing results before treatment and one month after surgery (standard deviation).

		Before Prehabilitation	One Month from Surgery	*p*
BMI (kg/m^2^)		28.54 (5.89)	30.29 (21.93)	0.316
	Underweight	7 (5.1)	4 (2.9)	0.456
BMI Categorized N (%)	Normal weight	43 (31.2)	46 (33.3)	
	Overweight	88 (63.8)	88 (63.8)	
Albumin (g/dL)		4.01 (1.95)	3.45 (0.07)	0.002
Prealbumin (mg/dL)		24.08 (6.89)	22.40 (5.15)	0.808
Proteins (g/dL)		6.97 (0.51)	5.95 (1.34)	0.477
Lymphocytes (uni/mm^3^)		1770.80 (762.55)	1833 (450.90)	0.888
Malnutrition (GLIM) (%)		64.80	26.70	<0.001
Dynamometry (kg)		24.06 (9.30)	27.21 (9.59)	0.007
Muscle mass (%)		33.54 (6.60)	36.69 (6.63)	<0.001
Fat mass (%)		33.45 (10.23)	30.28 (10.07)	0.011
Y QRF (cm)		1.16 (0.32)	1.37 (0.31)	<0.001
Y/(height^2^)		0.482 (0.156)	0.525 (0.187)	0.039
Preperitoneal fat thickness (cm)		0.680 (0.32)	0.500 (0.21)	<0.001
Barthel index N (SD)		96.90 (10.05)	96.98 (8.39)	0.951

**Table A7:** Clinical variables comparing results day before surgery and one month after surgery (standard deviation).

		Before Surgery	One Month from Surgery	*p*
BMI (kg/m^2^)		28.07 (5.46)	30.29 (21.93)	0.730
	Underweight	6 (4.3)	4 (2.9)	0.456
BMI Categorized N (%)	Normal weight	38 (27.5)	46 (33.3)	
	Overweight	94 (68.1)	88 (63.8)	
GLIM N (SD)		1.98 (1.44)	1.16 (1.31)	<0.001
Dynamometry (kg)		26.91 (9.76)	27.21 (9.59)	0.801
Muscle mass (%)		36.33 (6.45)	36.69 (6.63)	0.650
Fat mass (%)		31.03 (9.61)	30.28 (10.07)	0.532
Y QRF (cm)		1.40 (0.33)	1.37 (0.31)	0.444
Quality of Life N (SD)		5.17 (0.79)	5.27 (0.94)	0.384
Barthel index N (%)		96.90 (10.05)	96.98 (8.39)	0.951

**Table A8:** Quality of Life performance using the EuroQoL 5D test.

		SWL Inclusion N (%)	Before Surgery N (%)	One Month from Surgery N (%)
Walking Problems	Nothing	28 (20.3)	138 (100)	138 (100)
	Same	110 (79.9)	0	0
	A lot	0	0	0
Personal care problems	Nothing	138 (100)	138 (100)	138 (100)
	Same	0	0	0
	A lot	0	0	0
Daily activity problems	Nothing	0	138 (100)	138 (100)
	Same	111 (80.40)	0	0
	A lot	27 (19.60)	0	0
Pain	Nothing	0	111 (80.40)	138 (100)
	Same	83 (60.10)	27 (19.60)	0
	A lot	55 (39.90)	0	0
Anxity or depression	Nothing	28 (20.30)	138 (100)	138 (100)
	Same	110 (79.70)	0	0
	A lot	0	0	0

**Table A9:** Clinical variables comparing the results of patients who had post-operative complications with those who did not (standard deviation).

	Complications	No Complications
BMI (kg/m^2^)	26.19 (5.5)	25.14 (3.4)
Dynamometry (kg)	26.83 (9.48)	29.72 (10.45)
Muscle mass (%)	36.10 (6.6)	39.82 (5.57)
Fat mass (%)	31.50 (9.9)	22.25 (8.0)
Y QRF (cm)	1.27 (0.27)	1.38 (0.32)
Preperitoneal fat thickness (cm)	0.60 (0.36)	0.50 (0.20)
Barthel index	96.25 (9.39)	97.20 (7.90)

**Table A10:** Clinical variables comparing patients who did not undergo pre-surgical prehabilitation with those who did benefit from the program.

		Control (*n* = 76)	Study (*n* = 138)	Total (*n* = 214)	*p*
Complications *n* (%)	Yes	40 (52.60%)	19 (14.20%)	59 (27.60%)	<0.001
	No	36 (47.40%)	119 (85.80%)	155 (72.40%)	
Suture deshicense *n* (%)	Yes	16 (21.10%)	4 (3.00%)	20 (9.30%)	0.005
	No	60 (78.90%)	134 (97.00%)	194 (90.70%)	
Blood requierements *n* (%)	Yes	19 (25%)	13 (9.90%)	32 (15.50%)	0.014
	No	57 (75.00%)	122 (90.10%)	179 (84.5%)	
Length of Stay Days (SD)		11.63 (10.63)	8.34 (6.70)	9.50 (8.40)	0.004
